# Prognostic value of GLUT-1 expression in pancreatic cancer: results from 538 patients

**DOI:** 10.18632/oncotarget.15035

**Published:** 2017-02-02

**Authors:** Gaowa Sharen, Yaojun Peng, Haidong Cheng, Yang Liu, Yonghong Shi, Jian Zhao

**Affiliations:** ^1^ Cancer Center Key Lab, Chinese PLA General Hospital & Chinese PLA Medical School, Beijing 100853, P. R. China; ^2^ Molecular Pathology Laboratory, College of Basic Medicine, Inner Mongolia Medical University, Inner Mongolia 010059, P. R. China; ^3^ Department of General Surgery, The First Affiliated Hospital of Inner Mongolia Medical University, Inner Mongolia 010059, P. R. China; ^4^ Department of Endocrine, Chinese PLA 309 Hospital, Beijing 100071, P. R. China; ^5^ Department of Pathology, The Affiliated Hospital of Inner Mongolia Medical University, Inner Mongolia 010059, P. R. China; ^6^ International Joint Cancer Institute, The Second Military Medical University, Shanghai 200433, P. R. China

**Keywords:** pancreatic cancer, biomarker, meta-analysis, clinical, epidemiology

## Abstract

**Objective:**

Previous studies have suggested a correlation between glucose transporter-1 (GLUT-1) expression and survival outcomes in pancreatic cancer, although the results were inconsistent. We subsequently carried out a meta-analysis, with the aim of comprehensively reevaluating the associations between GLUT-1 expression and overall survival (OS) and other clinical features of pancreatic cancer.

**Results:**

Eight studies, with a total of 538 cases, were included in the final meta-analysis. The HR and 95% CI for OS were 1.79 and 1.19-2.7, respectively (p=0.005). GLUT-1 overexpression was associated with tumor size (>2 cm vs. ≤2 cm; OR=2.16, 95% CI=1.2-3.9, p=0.01) and lymph node metastasis (yes vs. no; OR=3.29, 95% CI=1.38-7.84, p=0.007). However, there was no significant association between GLUT-1 expression and histological grade, age, sex, TNM stage, or vascular invasion status. There was no evidence of significant publication bias in this meta-analysis.

**Materials and Methods:**

Relevant databases were searched using predefined searching items until September 2016. The pooled hazard ratios (HR) with 95% confidence interval (CI) for OS and the pooled odds ratio (OR) with 95% CI for clinical factors were calculated.

**Conclusions:**

High GLUT-1 expression predicted shorter OS in patients with pancreatic cancer. Moreover, GLUT-1 expression was associated with a tumor size of >2 cm and presence of lymph node metastasis.

## INTRODUCTION

Pancreatic cancer has a high mortality rate among all cancer types [1]. Of the 10 leading types of cancer in the United States, pancreatic cancer has a morbidity of approximately 3%, while the total mortality due to pancreatic cancer is approximately 7%[2]. The 5-year survival rate of patients with pancreatic cancer is 6%, and only one-fifth of all patients are eligible for curative surgery at the time of first diagnosis [3]. The very poor prognosis of this disease is largely attributed to early locoregional invasion and distant metastasis [4]. Tumor differentiation and size, and carbohydrate antigen 19-9 are independent prognostic factors that are often used to predict survival; however, these biomarkers lack sensitivity or specificity for prognostication [5]. In order to be able to make more individualized therapeutic regimens for patients, it is important to identify novel and valid biomarkers for pancreatic cancer.

Increased glycolytic metabolism is an important characteristic of cancer cells [6]. Glucose transporters (GLUTs) are a family of proteins containing 13 members that mediate glucose transport through the membrane into cells [7, 8]. Glucose transporter-1 (GLUT-1) is the first established member of GLUT family, which is normally expressed in erythrocytes, renal tubules, and the placenta [9]. In recent years, GLUT-1 was also reported to be overexpressed in various cancer types including hepatocellular carcinoma [10], prostate carcinoma [11], lung adenocarcinoma [12], oral squamous cell carcinoma [13], and pancreatic cancer [14–16]. Interestingly, previous studies provided conflicting results regarding the correlation of GLUT-1 expression and clinical outcomes in pancreatic cancer [14, 17, 18]. For example, Kitasato et al. identified GLUT-1 overexpression as being a significant biomarker for overall survival (OS) in pancreatic cancer patients (p=0.0102)[18]. Pizzi et al. also reported the independent prognostic role of GLUT-1 in pancreatic cancer by multivariate analysis [16]. However, Basturk and colleagues failed to find a significant prognostic function of GLUT-1 in their study (p=0.8392)[14]. Furthermore, Lyshchik et al. reported non-significant results (p=0.29) concerning the prognostic role of GLUT-1 in pancreatic cancer [19]. We therefore conducted a meta-analysis of eligible studies to quantitatively assess the association between GLUT-1 expression and OS as well as clinical features in patients with pancreatic cancer.

## RESULTS

### Study selection process

**Figure 1:**
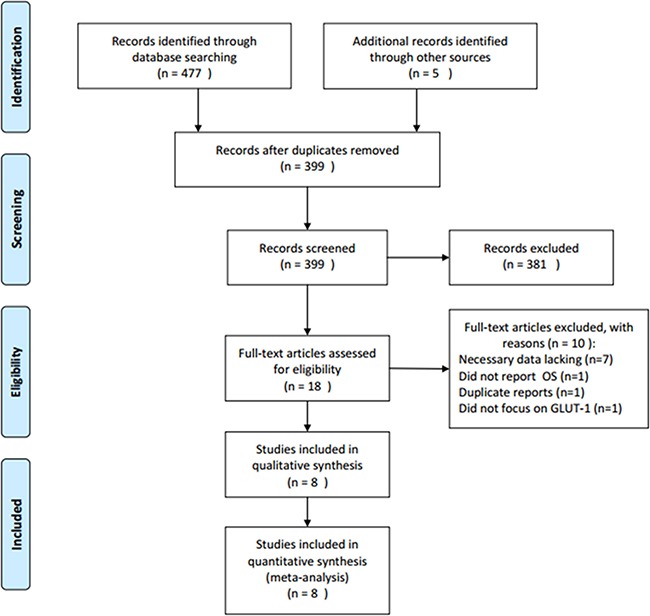
Flow chart of study selection for the meta-analysis

Figure 1 shows the literature selection procedures, where a total of 482 records were identified after the initial search procedure. After duplicate records were removed, 399 studies were screened by reviewing either title and/or abstract. A total of 381 records were subsequently excluded, and 18 full-text articles were further evaluated. Ten full-text articles were excluded because they did not provide necessary data such as OS, were duplicate studies, or did not focus on GLUT-1. A final total of eight studies were eligible for this meta-analysis [14–21].

### Study characteristics

Of the eight included studies, three [17, 20, 21] were from China, two from Japan [18, 19], and one each from the USA [14], Italy [16], and Korea [15]. All included studies made use of immunohistochemistry (IHC) to detect GLUT-1 expression, and all reported data on OS. Five studies [14, 15, 17, 20, 21] reported the data on GLUT-1 expression and clinical factors. The studies were published from 2007 to 2016. The sample sizes ranged from 41 to 106, and the total sample size was 538. The main characteristics of included studies are listed in Table 1.

**Table 1:** Characteristics of included studies

First author	Year	Country	Ethnicity	Age	Sample size	Stage	Treatment	Method	Survival analysis	HR (95%CI)	NOS score
Basturk	2011	USA	Caucasian	NR	94	I-IV	Surgery	IHC	OS	0.84(0.8-1.2)	7
Kitasato	2014	Japan	Asian	median 65	41	I-IV	Surgery	IHC	OS	2.62(1.26-5.47)	8
Lu	2016	China	Asian	median 63	53	I-II	Surgery	IHC	OS	3.4(1.76-6.54)	8
Lyshchik	2007	Japan	Asian	mean 63.5	74	I-IV	Surgery	IHC	OS	1.28(0.81-2.03)	7
Pizzi	2009	Italy	Caucasian	mean 65.4	60	I-IV	Surgery	IHC	OS	2.81(1.1-8)	7
Sun	2007	China	Asian	median 62	58	I-IV	Surgery	IHC	OS	2.23(1.22-4.05)	8
Sung	2010	Korea	Asian	median 62	52	I-IV	Surgery	IHC	OS	1.64(0.95-2.83)	7
Yu	2015	China	Asian	median 61	106	I-II	Surgery	IHC	OS	2.06(1.3-3.25)	8

NR: not reported.

### GLUT-1 and OS in pancreatic cancer

**Figure 2:**
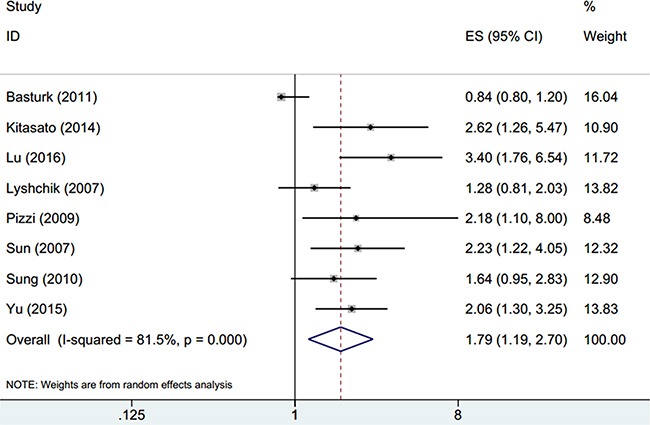
Forest plot for the association between GLUT-1 expression and OS in pancreatic cancer

Eight studies [14–21] evaluated OS considering GLUT-1 expression. Owing to significant heterogeneity in pancreatic cancer cases in the eight studies (*I*^2^=81.5%, P_h_<0.001), a random-effects model was used, with the resulting overall HR and 95% CI being 1.79 and 1.19-2.7, respectively (p=0.005; Figure 2, Table 2). Subgroup analysis indicated that GLUT-1 expression remained a significant biomarker for OS in both stages I-IV and I-II (Table 2). However, expression of GLUT-1 still predicted poor OS in Asian individuals (HR=1.94, 95% CI=1.55-2.43, p<0.001; Table 2), whereas GLUT-1 expression was not a significant biomarker for poor OS in Caucasian individuals (HR=1.19, 95% CI=0.48-2.93, p=0.705; Table 2).

**Table 2:** Stratified analysis of pooled HRs for GLUT-1 and OS in pancreatic cancer

variable	No. of studies	HR (95% CI)	p	Heterogeneity		Model used
				I^2^(%)	P_h_	
OS	8	1.79(1.19-2.7)	0.005	81.5	<0.001	Random
Ethnicity						
Asian	6	1.94(1.55-2.43)	<0.001	30.9	0.204	Fixed
Caucasian	2	1.19(0.48-2.93)	0.705	70.7	0.065	Random
Stage						
I-IV	6	1.56(1.01-2.4)	0.045	77.1	0.001	Random
I-II	2	2.43(1.67-3.53)	<0.001	33.6	0.22	Fixed

### GLUT-1 and clinical features in pancreatic cancer

Five studies [14, 15, 17, 20, 21] investigated the association between GLUT-1 expression and tumor size (odds ratio [OR]=2.16, 95% CI=1.2-3.9, p=0.01; Table 3) and lymph node metastasis (OR=3.29, 95% CI=1.38-7.84, p=0.007; Table 3). We also analyzed the associations between GLUT-1 expression, and histological grade, age, sex, TNM stage, and extent of vascular invasion. The combined data (Table 3) indicated that the associations between GLUT-1 and the following parameters were not significant: histological grade (OR=1.95, 95% CI=0.74-5.16, p=0.18), age (OR=0.95, 95% CI=0.58-1.57, p=0.855), sex (OR=1, 95% CI=0.66-1.82, p=0.712), TNM stage (OR=1.69, 95% CI=0.44-6.52, p=0.449), and vascular invasion (OR=0.58, 95% CI=0.27-1.22, p=0.149).

**Table 3:** Correlation of GLUT-1 and clinical factors in pancreatic cancer

Variable	No. of studies	OR (95% CI)	p	Heterogeneity		Model used
				I^2^ (%)	P_h_	
Tumor size (>2 cm vs ≤2 cm)	5	2.16(1.2-3.9)	0.01	41.2	0.147	Fixed
Lymph node metastasis (yes vs no)	5	3.29(1.38-7.84)	0.007	64.4	0.024	Random
Histological grade (poor vs moderate/well)	4	1.95(0.74-5.16)	0.18	61.8	0.049	Random
Age (>62 vs ≤62)	4	0.95(0.58-1.57)	0.855	0	0.57	Fixed
Gender (male vs female)	4	1(0.66-1.82)	0.712	0	0.947	Fixed
TNM stage (III + IV vs I + II)	3	1.69(0.44-6.52)	0.449	71.5	0.03	Random
Vascular invasion (yes vs no)	3	0.58(0.27-1.22)	0.149	0	0.437	Fixed

### Publication bias

**Figure 3:**
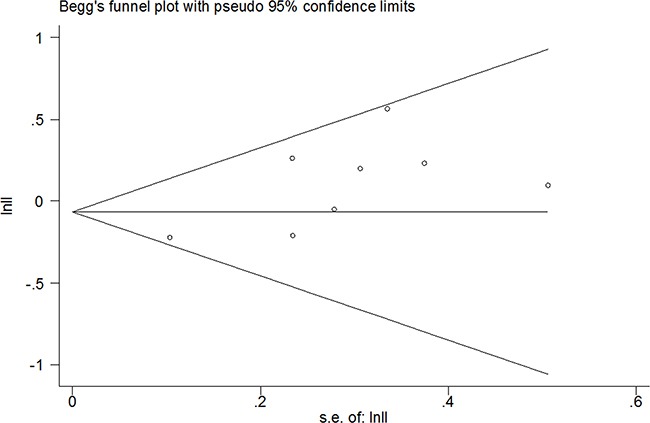
Publication bias using Begg's test for OS (p=0.386)

Publication bias was determined by using the Begg's test. The results showed that Begg's p-value was 0.386 for GLUT-1 and OS analysis (Figure 3). The data demonstrated that there was no evidence of significant publication bias in this meta-analysis.

## DISCUSSION

Pancreatic cancer has poor prognosis, with a 5-year survival rate of <5%, owing to the fact that most cases are asymptomatic until the disease proceeds to advanced stages [5]. Several prognostic biomarkers are currently applied in the clinical management of pancreatic cancer; however, the prognostic efficiency is limited [4]. In the present study, we performed a meta-analysis that demonstrated that GLUT-1 expression was associated with shorter OS in patients with pancreatic cancer. In addition, GLUT-1 was also correlated with larger tumor size (>2 cm) and presence of lymph node metastasis. Taken together, these results demonstrated that GLUT-1 overexpression has the potential to be a biomarker for poor survival and dissemination in pancreatic cancer. To the best of our knowledge, the present study was the first meta-analysis to date to show the prognostic significance of GLUT-1 in pancreatic cancer.

GLUT-1 is the most common glucose transporter in humans, which is also widely expressed in a variety of malignant tumors [6, 22]. High GLUT-1 expression was reported to be related with pancreatic cancer invasiveness, and GLUT-1 was also implicated in a pancreatic carcinogenesis progression model [14, 23]. GLUT-1 overexpression in tumor tissues reflects the increased requirement for glucose, which is one of the main fuel sources for cancer cells. GLUT-1 was found to be correlated with biological behaviors of different tumors [24, 25]. In addition, GLUT-1 overexpression was also found to be associated with cancer progression and poor survival outcomes in different types of cancer, including bone and soft-tissue sarcomas [26], colorectal cancer [27], breast cancer [28], and lung cancer [29]. The findings of the present meta-analysis were in line with the results of previous studies on other types of cancer. More importantly, several primary studies [14, 15, 19] on pancreatic failed to find the positive correlation between GLUT-1 overexpression and poor survival in pancreatic cancer. For example, both Basturk et al. [14] and Lyshchik [19] failed to identify GLUT-1 expression as a significant marker for OS (p=0.8392 and p=0.29, respectively). By contrast, other investigators [16, 18, 20, 21] showed the prognostic value of GLUT-1 expression for pancreatic cancer. The discrepancies may be owing to the following reasons: firstly, different antibodies were used in IHC, and confounding factors such as differences in brand, isotype, and dilution could lead to heterogeneity of results. Secondly, patient selection criteria differed between the eight studies, and these patients were of different ethnic backgrounds. Third, the follow-up in included studies were different, which may also have had an impact on the individual results. Our meta-analysis pooled the conflicting results from eligible studies and presented a relatively definitive conclusion after quantitative calculations. Therefore, this meta-analysis provided implications for clinical practice in pancreatic cancer.

However, several limitations of the present meta-analysis need to be acknowledged. Firstly, only studies published in English were included in the final meta-analysis, and while both English and Chinese papers were searched, the eight eligible studies (according to inclusion and exclusion criteria) were all published in English. Secondly, the sample size of articles was relatively small, where only eight studies with 538 cases in total were included, which may have introduced selection bias. Lastly, there were limited categories where subgroup analyses could be performed, because studies were selected according to uniform inclusion criteria, and the eligibility criteria were therefore homogeneous in some aspects.

In conclusion, our study demonstrated that increased GLUT-1 expression was significantly associated with poor OS in pancreatic cancer. Moreover, GLUT-1 expression was also significantly correlated with tumor size of >2 cm and presence of lymph node metastasis. Owing to the limitations mentioned above, more large-scale studies are needed to confirm the validity of GLUT-1 expression as a prognostic biomarker in pancreatic cancer.

## MATERIALS AND METHODS

### Literature search strategy

The current meta-analysis was carried out according to the Preferred Reporting Items for Systematic Reviews and Meta-Analyses (PRISMA) statement [30]. The following electronic databases were comprehensively searched for relevant studies: PubMed, Web of Science, Embase, and China National Knowledge Infrastructure (CNKI). The last search was updated on September 15, 2016. The search terms were as follows: (glucose transporter-1 or GLUT-1 or glut1 or GLUT or glucose transporter or SLC2A1) and (pancreatic or pancreas*) and (cancer or tumor or tumour or neoplasm or carcinoma or adenocarcinoma) and (prognosis or prognostic or predict or predictive or treatment or survival). References of all relevant articles were also screened to find other potentially applicable studies.

### Selection criteria

Papers published in either English or Chinese were eligible for selection, and the inclusion criteria were as follows: 1) the diagnosis of pancreatic cancer was established on anatomical pathology specimen examination; 2) GLUT-1 expression was detected by immunohistochemistry (IHC) in tissue specimens; 3) studies must have reported the hazard ratio (HR) and 95% confidence interval (CI) for OS or provided sufficient information for calculation according to Tierney's method [31] and 4) studies were published as full-text articles. If different studies were published based on the same patients group, then the more comprehensive one was selected. Exclusion of studies was based on the following criteria: 1) meeting abstracts, reviews, case reports, and letters; 2) duplicate studies; 3) animal studies; and 4) studies with insufficient information. Two investigators independently evaluated the eligibility of relevant studies per selection criteria, and discrepancies were resolved by discussion.

### Data extraction and quality assessment

The following information was extracted from included studies: first author, year, country, number of patients, treatment, tumor stage, detection method, age, and survival data. If any data was lacking, they were labeled as “not reported”. The quality of eligible studies was evaluated by using Newcastle-Ottawa Quality Assessment Scale (NOS). The NOS scale was composed of three categories with a full score of 9 stars. Studies scored ≥6 stars were considered to be of high quality.

### Statistical analysis

HR and 95% CI were used to estimate the impact of GLUT-1 on OS. Cochran's Q test and *I*^2^ were used to assess the heterogeneity of the included studies. A p-value of <0.10 for Cochrane's Q test or *I*^2^>50% represented significant heterogeneity, and the random-effects (DerSimonian–Laird method) model was subsequently used in these analyses. The fixed-effects (Mantel–Haenszel method) model was utilized for the remaining analyses. Subgroup analysis was conducted for exploring heterogeneity and for further analysis. ORs and 95% CIs were used as effective measure of the association between GLUT-1 expression and clinical features in pancreatic cancer, while publication bias was estimated by using Begg's funnel plot (p<0.05 was considered statistically significant). All analyses were performed by using STATA software version 12.0 (StataCorp LP, College Station, TX, USA).
